# Prevention and treatment strategies for kidney transplant recipients in the context of long-term existence of COVID-19

**DOI:** 10.3389/fmed.2024.1287836

**Published:** 2024-04-03

**Authors:** Zeyuan Zheng, Huimin Sun, Xiaoyan Hu, Zuodong Xuan, Meiling Fu, Yang Bai, Yifan Du, Bin Liu, Xiuyuan Sui, Jianzhong Zheng, Chen Shao

**Affiliations:** ^1^Department of Urology, Xiang’an Hospital of Xiamen University, School of Medicine, Xiamen University, Xiamen, China; ^2^Central Laboratory, Xiang’an Hospital of Xiamen University, School of Medicine, Xiamen University, Xiamen, China

**Keywords:** Omicron, kidney transplant recipients, drugs, vaccines, treatment

## Abstract

The sudden outbreak of coronavirus disease 2019 (COVID-19) in early 2020 posed a massive threat to human life and caused an economic upheaval worldwide. Kidney transplant recipients (KTRs) became susceptible to infection during the COVID-19 pandemic owing to their use of immunosuppressants, resulting in increased hospitalization and mortality rates. Although the current epidemic situation is alleviated, the long-term existence of COVID-19 still seriously threatens the life and health of KTRs with low immunity. The Omicron variant, a highly infectious but less-pathogenic strain of severe acute respiratory syndrome coronavirus 2 (SARS-CoV-2), has raised concerns among transplant physicians regarding managing KTRs diagnosed with this variant. However, currently, there are no clear and unified guidelines for caring for KTRs infected with this variant. Therefore, we aimed to summarize the ongoing research on drugs that can treat Omicron variant infections in KTRs and explore the potential of adjusting immunotherapy strategies to enhance their responsiveness to vaccines. Herein, we discuss the situation of KTRs since the emergence of COVID-19 and focus on various prevention and treatment strategies for KTRs since the Omicron variant outbreak. We hope to assist physicians in managing KTRs in the presence of long-term COVID-19 variants.

## Graphical abstract

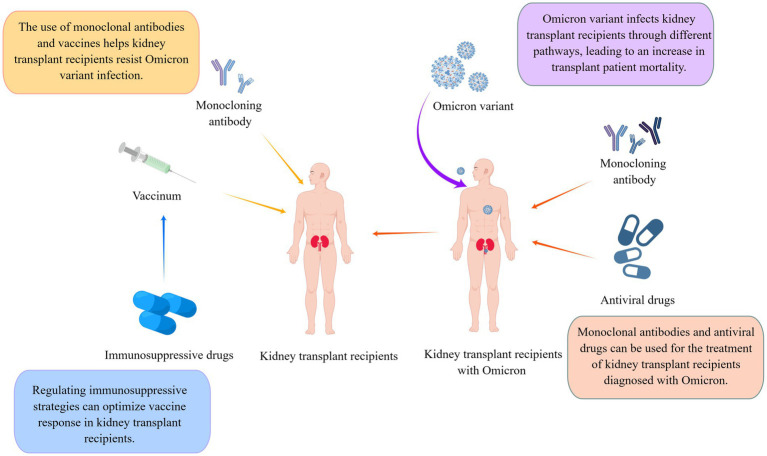

## Highlights

- Monoclonal antibodies help kidney transplant recipients resist Omicron infection;
- Antiviral drugs can be used for the treatment of kidney transplant recipients diagnosed with Omicron;
- Alternative vaccination strategies can optimize the vaccination response of kidney transplant recipients;
- Regulating immunosuppressive strategies to optimize vaccine response in kidney transplant recipients.

## 1 Introduction

Organ transplantation is recognized as the most efficacious therapeutic approach for individuals afflicted by end-stage organ failure (1). Among all organ transplantations, kidney transplantation is the earliest, most common, and most widely used with the best outcomes (2). Chronic kidney disease (CKD) afflicts an estimated 700 million individuals worldwide and constitutes a formidable peril to patients’ lives when it advances to end-stage kidney disease (ESKD), resulting in the annual mortality of roughly 5–10 million individuals worldwide (3). Kidney transplantation is regarded as the optimal therapeutic intervention for ESKD patients, remarkably enhancing their long-term survival prospects, affording an improved quality of life, and yielding greater cost-effectiveness compared with dialysis therapy (4, 5).

Coronavirus disease 2019 (COVID-19) was brought about by severe acute respiratory syndrome coronavirus 2 (SARS-CoV-2), also known as novel coronavirus, rapidly evolving into a worldwide pandemic in early 2020, with its impact extending to a minimum of 222 countries and territories worldwide (6). Due to pre-existing CKD and long-term immunosuppressive therapy, kidney transplant recipients (KTRs) were found to be highly vulnerable to COVID-19 infection (7). Following the initial report of a KTR with COVID-19 pneumonia in March 2020, the incidence of COVID-19 in KTRs and its various strains has been on the rise.

The Omicron variant boasts the highest number of mutations among all the SARS-CoV-2 variants. The variant XBB and its descendant lineage EG.5(Eris) are important reasons for the current pandemic. Due to its heightened transmissibility and capacity to evade the immune response, Omicron has swiftly overridden the Delta variant and emerged as the prevailing variant in numerous regions worldwide (8). Managing KTRs during the Omicron variant epidemic is a significant concern for urologists. Although some monoclonal antibodies and antiviral drugs have been approved for use in KTRs during SARS-CoV-2 and other subtype outbreaks, their effectiveness against the Omicron variant in KTRs remains to be ascertained. Moreover, owing to the unique immunosuppressive state of KTRs, the impact of prior vaccination on protecting KTRs from Omicron variant attacks is unclear. A more comprehensive vaccine system is required to help immunocompromised patients fight the Omicron variant. This review aims to offer guidance and a point of reference for the care and management of KTRs in the ongoing presence of COVID-19 and its different variants. This is achieved by consolidating pertinent clinical research and treatment strategies related to KTRs amidst the prevalence of the Omicron variant.

## 2 Kidney transplantation and COVID-19

Since the onset of the COVID-19 pandemic, the rate of SARS-CoV-2 infection in KTRs has consistently exceeded that observed in the general population (9). Allograft rejection indicates that KTRs must use long-term immunosuppressants such as prednisone and mycophenolate mofetil (MMF) to prevent rejection risk. However, extended use of immunosuppressants, along with comorbid conditions such as hypertension and diabetes, and the reduced vaccine response in KTRs, considerably increase their susceptibility to COVID-19 (10). In addition to increasing the difficulty of treatment and care for KTRs with underlying COVID-19 infection, the virus can cause damage to the transplanted kidney (11). Furthermore, compared with non-transplant patients, KTRs have a higher frequency of acute kidney injury (AKI; 27.5% vs. 13.3%) and a more frequent need for renal replacement therapy (15.4% vs. 3.3%) (7, 12, 13). The mortality rate of patients with kidney failure and KTRs was observed to be particularly high after COVID-19 infection (14). This conclusion is consistent with a data report in the United States, according to which, after accounting for comorbidities, mortality risk for transplant recipients was almost twice as high as that for non-transplant patients (15).

**Figure 1:**
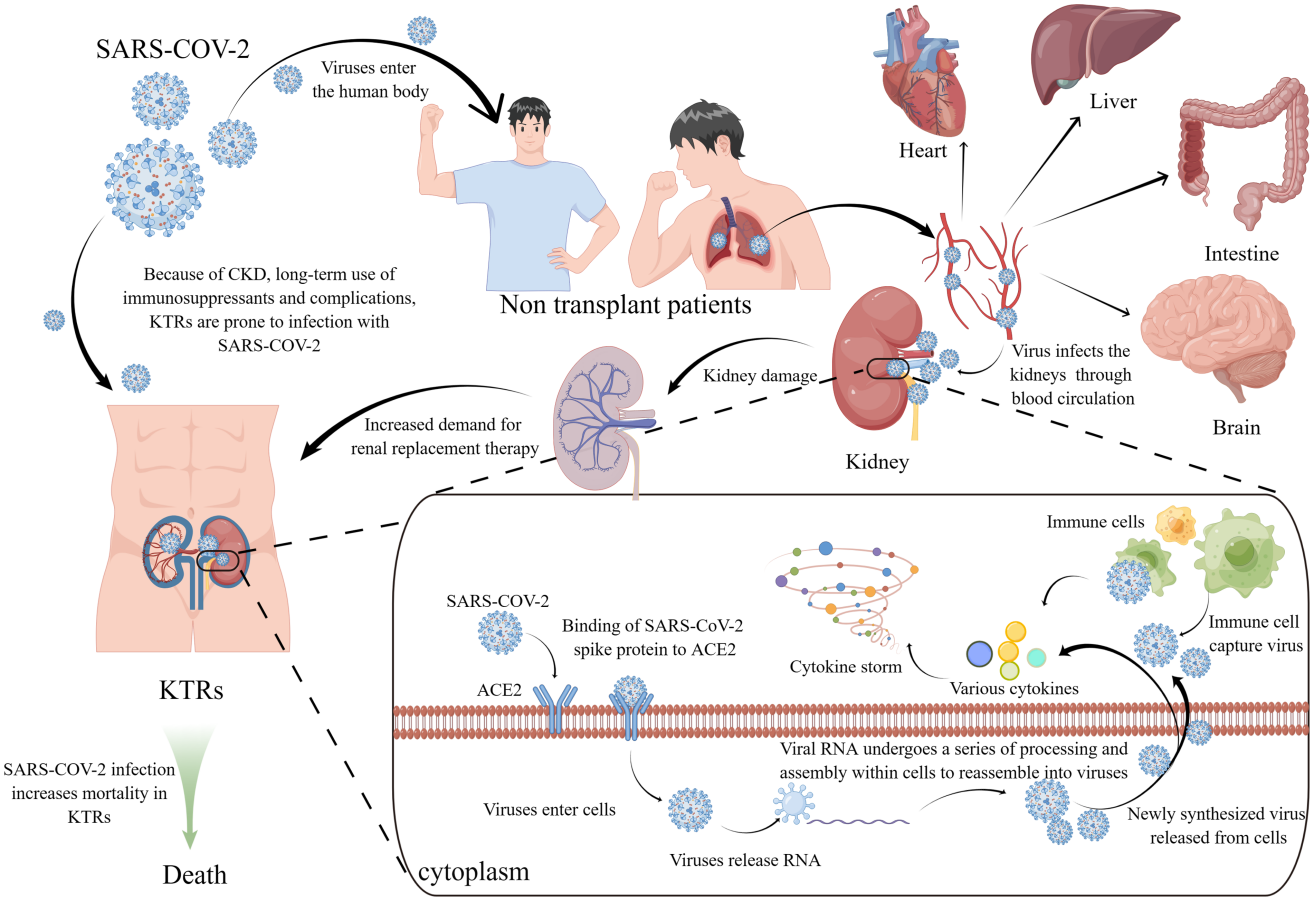
KTRs and SARS-COV-2 and its infection mechanism. KTRs are susceptible to SARS COV-2 due to CKD and many complications. After infection with SARS-COV-2, the mortality of KTR has increased significantly. SARS-COV-2 enters the human body through the respiratory tract, gathers in the lungs, and then spreads to various organs (including the heart, liver, brain, kidney, etc.) through the blood. The viral spike protein of SARS-COV-2 combines with the ACE2 receptor of renal epithelial cells, and then enters the cells, releasing viral RNA. The viral RNA combines with the ribosome, translates, replicates, and other operations to assemble new viruses. The virus is released from the cells through exocytosis, At the same time, cytokines are also released, and subsequently immune cells aggregate and capture the virus, while also releasing a large number of cytokines. The cell storm formed by numerous cytokines and the virus itself damage cells, causing damage to the patient’s kidney and increasing the demand for kidney transplant treatment. KTRs, kidney transplant recipients; SARS-COV-2, Severe acute respiratory syndrome coronavirus 2; CKD, Chronic kidney disease; ACE2, angiotensin-converting enzyme 2.

The kidney stands as one of the specific organs susceptible to SARS-CoV-2 infection (Figure 1). After a patient is diagnosed with SARS-CoV-2 lung infection, the virus can enter the kidney through blood circulation and accumulate, damaging resident cells and causing kidney injury (16). SARS-CoV-2 can directly infect the human kidneys, leading to tubulopathy and AKI (17). SARS-CoV-2 pathogenesis closely resembles that of SARS and hinges on the interaction between the viral spike protein and the angiotensin-converting enzyme 2 (ACE2) receptor, with cell entry facilitated by the cellular protease TMPRSS2 (18). ACE2 receptors are widely distributed in multiple organs in the human body, with high expression in the kidney (19), particularly in the proximal tubules (20). After SARS-CoV-2 enters kidney cells, viral RNA is translated, inducing innate immune responses through the host’s expression of interferons, chemokines, and cytokines such as tumor necrosis factor (TNF), interleukin-1β (IL-1β), and interleukin-6 (IL-6) (21, 22). As the virus continues to replicate, cytokine levels continue to rise, leading to severe tissue damage and cytokine storms in patients (23), which can cause enormous damage to the kidneys (24). As the virus replicates, mutations in the spike protein’s receptor-binding domain (RBD) may occur, resulting in the emergence of new viral variants. These mutations increase the RBD’s affinity for the ACE2 complex, boosting the virus’s infectivity and transmission.

Since the advent of COVID-19, the World Health Organization (WHO) has identified five distinct variants of SARS-CoV-2, namely, Alpha (B.1.1.7), Beta (B.1.351), Gamma (P.1), Delta (B.1.617), and Omicron (B.1.1.529) (25). The B.1.1.529 variant initially surfaced in South Africa on November 9, 2021, and was later designated as the fifth variant by the WHO and given the name Omicron. By December 22, 2021, the Omicron variant had been identified in 110 countries and regions across the globe (26). Omicron then swiftly surpassed the Delta variant as the prevailing strain on a global scale. The former has been noted for its significantly enhanced transmissibility, although it exhibits a lower case fatality rate than both the Delta variant and the original SARS-CoV-2 virus (27). Despite the decrease in the case fatality rate associated with the Omicron variant when contrasted with earlier strains, a retrospective study conducted on KTRs in a specific region of Spain revealed that following infection with the Omicron variant, the occurrence of pneumonia and the mortality rate continued to surpass those observed in the general population (28). Furthermore, the Omicron variant increased the hospitalization rate of KTRs, possibly due to the increased renal burden caused by inflammation and tissue damage after infection with SARS-CoV-2 (29). Therefore, during the Omicron variant outbreak, KTRs, a vulnerable population, need enhanced focus on infection prevention and suitable treatments for those diagnosed with the variant. In summary, it is essential to conduct more research and create tailored prevention and treatment strategies for KTRs during the Omicron pandemic.

## 3 Application of monoclonal antibodies for treating KTRs infected with Omicron

SARS-CoV-2, a beta coronavirus, is an RNA virus. Similar to other viruses in this group, certain points in its lifecycle can be identified and blocked using monoclonal antibodies (mAbs), making them a promising option for preventing and treating COVID-19 (30). During the SARS-CoV-2 pandemic, mAbs have been approved for vulnerable populations, including KTRs (31). Nevertheless, with the Omicron variant’s emergence, there is uncertainty about the effectiveness of mAbs. Takashita et al. (32) assessed mAb reactivity via enzyme-linked immunosorbent assays. Results showed bamlanivimab lost its ability to neutralize Omicron, while imdevimab had high neutralization against Beta and Gamma variants but not Omicron. In contrast, caplacizumab, tixagevimab (COV2-2196), cilgavimab (COV2-2130), and sotrovimab precursor (S309) maintained neutralization against Beta, Gamma, and Omicron. However, their FRNT50 values for Omicron were 3.7 to 198.2 times higher than those for Beta or Gamma.

### 3.1 Sotrovimab

Sotrovimab is a recombinant human mAb designed to target SARS-CoV-2. Its mechanism of action involves neutralizing the virus by binding to a highly conserved epitope located on the RBD of the spike protein (33). Sotrovimab has demonstrated its effectiveness in treating COVID-19 in solid organ transplant recipients (SOTRs) (34). However, its efficacy against Omicron variants in KTRs remains to be validated.

A single-center study revealed favorable tolerability and efficacy characteristics of sotrovimab in transplant patients and preserved activity against the Omicron variant (35). Furthermore, individuals with the Omicron variant who received sotrovimab treatment demonstrated a reduced probability of hospitalization or clinic visits (36). Radcliffe et al. (37) analyzed 122 patients diagnosed with Omicron, including 24 who received sotrovimab treatment, 48 who did not receive outpatient treatment, and the remaining patients who received antiviral therapy. The patients were followed up for more than 30 days, and the hospitalization rate of patients who received sotrovimab treatment within 30 days was 8% (2/24), while that of patients who did not receive outpatient treatment was 27% (13/48), implying that sotrovimab has activity against the Omicron variant. Additionally, a single-center retrospective study conducted by Fernandes et al. (38) revealed that sotrovimab administration was linked to favorable outcomes among KTRs who contracted mild COVID in 2019, including those infected with the Omicron variant.

Although sotrovimab has been approved for adolescents and adults, its effectiveness and safety in KTRs under 12 years of age are unclear. Chiara et al. (39) described the case of an 11-year-old male KTR who got COVID-19 after a kidney transplant. Due to the patient’s kidney function, sotrovimab was administered, and the treatment was deemed successful without adverse effects. This case adds to the body of evidence on sotrovimab’s efficacy and safety in pediatric patients. However, it should be noted that the susceptibility of BA.2 subvariant of the Omicron variant to sotrovimab was diminished (40).

### 3.2 Bebtelovimab

In February 2022, bebtelovimab received authorization from the United States Food and Drug Administration (FDA) to treat COVID-19 (32). Recent studies have demonstrated that bebtelovimab maintains its effectiveness against the Omicron variant, including its subvariants BA.1.2, BA.4, and BA.5 (41). McCreary et al. (42) assessed the real-world effectiveness of bebtelovimab in outpatients with mild-to-moderate COVID-19 during the Omicron era. The authors concluded that using bebtelovimab for outpatient COVID-19 cases may reduce hospitalization and mortality risks, especially for the elderly and immunocompromised patients. However, it is unclear whether bebtelovimab is effective in immunocompromised groups such as KTRs.

Nonetheless, Shertel et al. (43) study addressed the above point and demonstrated that bebtelovimab can treat SOTRs with COVID-19 caused by the Omicron BA.1 or BA.25 subvariants. Their experimental results suggest that bebtelovimab could be a safe and effective treatment for transplant recipients with mild-to-moderate Omicron infections. However, this study was retrospective and lacked a control group; thus, the results may be less conclusive. Yetmar et al. (44) also explored the use of bebtelovimab in KTRs and found that, despite vaccination rates and viral subvariant differences as potential confounding factors, COVID-19 hospitalization rates were similar in patients treated with bebtelovimab and those treated with sotrovimab.

### 3.3 Tixagevimab/cilgavimab

Tixagevimab/cilgavimab, sold under the brand name Evusheld, is a monoclonal antibody drug that has received authorization from both the FDA and the UK Medicines and Healthcare Products Regulatory Agency. It was designed to reduce the risk of COVID-19. This treatment is intended for individuals who are presently uninfected or have not been exposed to the coronavirus and who exhibit a compromised immune response to COVID-19 vaccines. This includes individuals who are not advised to receive vaccinations (45). Consequently, individuals with compromised immune responses to vaccines, including KTRs, may consider tixagevimab/cilgavimab for pre-exposure prophylaxis and treating COVID-19 infections (46).

Tixagevimab/cilgavimab can mimic the natural antibody defense mechanism of the human body by binding to different sites on the spike protein of the novel coronavirus, reducing its ability to enter and infect healthy cells, and helping to clear infected cells (47). Medical practitioners have showcased the ability of this drug to reduce the risk of SARS-CoV-2 infection during the Alpha (B.1.1.7) and Delta (B.1.617.2) waves in unvaccinated individuals (48, 49). Following the emergence of the Omicron variant and its sub-lineages, tixagevimab/cilgavimab was recognized as the sole effective monoclonal combination therapy available (50), with neutralizing activity against the Omicron variant sub-lineages BA.1 and BA.1.1 (51)as well as against sub-lineages BA.2 and BA.5 (49).

Tixagevimab/cilgavimab is suitable for pre-exposure prophylaxis in KTRs because the binding domain of SARS-CoV-2 spike protein IgG titers of the recipient decreases rapidly after the recipient is administered this drug (52). A medical research team compared 222 SOTRs who received tixagevimab/cilgavimab for pre-exposure prophylaxis with 222 who did not. The team found that 5% of those receiving the drug had breakthrough SARS-CoV-2 infections, significantly lower (*p* < 0.001) compared with 14% in the control group. The results suggested that administering tixagevimab/cilgavimab to SOTRs who were vaccinated during the Omicron surge was safe and mitigated the risk of breakthrough SARS-CoV-2 infections (53).

While tixagevimab/cilgavimab may help prevent KTRs from being infected with the Omicron variant, further experiments are needed to determine the appropriate dosage, as Benotmane et al. (54) suggested that using a dose of 150 mg of each antibody may not provide sufficient protection against Omicron infection. Consequently, it is crucial to establish the required dosage for achieving neutralizing titers against various Omicron sub-lineages. Further research is essential to examine the pharmacological effects and antibody kinetics of the drug to provide valuable insights for the subsequent steps in treatment planning. However, tixagevimab/cilgavimab does not exhibit neutralizing activity against the Omicron sub-variant, BQ.1.1, and additional research is therefore warranted to assess its efficacy against this variant (55).

### 3.4 Other monoclonal antibodies

Dhand et al. (56) reported using casirivimab/imdevimab for COVID-19 treatment in SOTRs, which could prevent further progression of COVID-19 in KTRs to a certain extent (57). In addition to the results of sotrovimab administration, Fernandes et al. (38) showcased the positive outcomes of casirivimab/imdevimab administration in KTRs infected with the Omicron variant. However, the latest research shows that the inhibitory concentration (IC_50_) of casirivimab/imdevimab against the Omicron variant is >9000 ng/mL (58). Furthermore, Hoffmann et al. (59) found that Omicron exhibits complete resistance to imdevimab and casirivimab mAbs. Therefore, whether KTRs can benefit from casirivimab/imdevimab still requires further in-depth research, although it can provide at least one treatment option for clinical doctors.

Tocilizumab, a mAb that blocks the IL-6 receptor and acts by reducing inflammation, has been demonstrated to treat infectious complications in KTRs (60). Early administration of tocilizumab during the COVID-19 pandemic was found to decrease the mortality rate among severely ill COVID-19 patients (61). However, no research has explored whether tocilizumab is beneficial for KTRs diagnosed with the Omicron variant.

Takashita et al. (32) *in vitro* experiments revealed that mAbs such as LY-CoV555 (sold as bamlanivimab), LY-CoV016 (sold as etesevimab), and REGN10987 (sold as imdevimab) that originally acted on other variants lost their neutralizing effect against Omicron. This also limits the selection of mAbs that can be applied to treat KTRs diagnosed with the Omicron variant. In addition, many mAbs, such as BI 767551 (DZIF-10c) and CT-P59 (regdanvimab), have been approved for emergency use in treating COVID-19 (30, 62). However, only a few studies have attempted to ascertain whether these antibodies are suitable for immunocompromised patients.

To sum up, although mAbs have been instrumental in preventing and treating COVID-19, the effectiveness of current mAbs against evolving viruses such as Omicron has waned. Additionally, the distinctive circumstances of KTRs, coupled with limited research on mAb use in this group, have resulted in a shortage of suitable mAbs for this population (Table 1).

**Table 1:** Monoclonal antibodies for the treatment of COVID-19.

Monoclonal antibody	Targeted strain	Targeting Omicron subtypes	Inhibition target	Administration method	Authorization time	Application in KTRs	Reference
Sotrovimab (S309)	Beta variant, Gamma variant, Omicron variant.	Effective neutralization BA. 1; For BA. 2, BA. 4, and BA. 5, the efficacy decreased; no effect on BQ.1.1 or XBB.	SARS-CoV-2 Spike protein	iv	May 26, 2021, FDA has granted EUA to Sotrovimab	Yes	MacGregor et al. (63) and Nathalie et al. (64)
Bebtelovimab (LY-CoV1404)	SARS-CoV-2, Alpha variant, Beta variant, Delta variant, micron variant.	Effective neutralization BA.1,BA4,BA5, BA.4.6, BA.2.75.2; no effect on BQ.1.1 or XBB.	SARS-CoV-2 Spike protein	iv	February 11, 2022, FDA has granted EUA to Bebtelovimab. Bebtelovimab was no longer authorized after 30 November 2022	Yes	Kathryn et al. (65) and Zachary et al. (44)
Regdanvimab (CT-P59)	Beta variant, Delta variant.	—	SARS-CoV-2 Spike protein	iv	EC has approved Regdanvimab for the treatment of COVID-19 patients, not yet approved by FDA	Reported in HSCT patients, not yet reported in KTRs	Shinwon et al. (66), Subramaniam et al. (67), and Yu-Chyi et al. (30)
Adintrevimab (ADG20)	SARS-CoV-2, Alpha variant, Beta variant, Gamma variant, Delta variant.	Reduced *in vitro* activity of BA. 1 and BA. 1.1; Lack of activity towards BA. 2, BA. 3, BA. 4, BA. 5, BQ.1.1, and XBB	SARS-CoV-2 Spike protein	im	Currently not authorized by FDA	No	Elizabeth et al. (68) and Bruel et al. (69)
Tocilizumab	—	—	IL6	iv	December 21, 2022, FDA authorized tocilizumab for the treatment of COVID-19	Yes	Sylweste et al. (70)
BI 767551 (DZIF-10c)	SARS-CoV-2, Alpha variant, Beta variant	—	SARS-CoV-2 Spike protein	inhal	Preclinical, not authorized by the organization	No	Sandro et al. (71)

KTRs, kidney transplant recipients; SARS-CoV-2, Severe acute respiratory syndrome coronavirus 2; IL6, interleukin 6; iv, IntraVenous; inhal:inhalation; im, intramuscular injection; EC, European Commission; FDA, the United States Food and Drug Administration; EUA, Emergency-Use-Administration; HSCT, hematopoietic stem cell transplantation; COVID-19, Corona Virus Disease 2019.

Furthermore, the emergence of the latest Omicron sub-variants BQ.1.1 and XBB has made many mAbs that were previously beneficial to KTRs ineffective, such as imdevimab, casirivimab, tixagevimab, cilgavimab, bebtelovimab, and S309 (the precursor of sotrovimab) (55). Therefore, more comprehensive investigations must be conducted to verify the efficacy of existing antibodies or develop new antibodies. The combined use of mAbs is also an important and viable approach (Table 2).

**Table 2:** Monoclonal antibody combinations for the treatment of COVID-19.

Monoclonal antibody	Targeted strain	Targeting Omicron subtypes	Inhibition target	Administration method	Authorization time	Application in KTRs	Reference
Imdevimab (REGN10987) -Casirivimab (REGN10933)	SARS-CoV-2, Alpha variant, Beta variant, Delta variant, Omicron variant	Effectively neutralize BA. 1, BA. 2, BA. 4, and BA. 5; Poor neutralization effect on BA. 4.6; No effect on BQ.1.1 or XBB	SARS-CoV-2 Spike protein	iv	November 22, 2020, FDA has granted EUA to this antibody combination.	Yes	Esther et al. (72) and Abhay et al. (56)
Tixagevimab (COV2-2196) -Cilgavimab (COV2-2130)	SARS-CoV-2, Alpha variant, Beta variant, Delta variant, Omicron variant	Effectively neutralize BA. 10 effect on BQ.1.1 or XBB	SARS-CoV-2 Spike protein	im	December 8, 2021, FDA has granted EUA to this antibody combination.	Yes	Myron et al. (47) and Daniele et al. (48)
Etesevimab (LY-CoV016) -Bamlanivimab (LY-CoV555)	Alpha variant, Beta variant, Gamma variant	—	SARS-CoV-2 Spike protein	iv	FDA has granted EUA to this antibody combination.	Yes	Michael et al. (73) and Heather et al. (74)
Amubarvimab (BRII-196) -Romlusevimab (BRII-198)	Omicron variant	Effective Neutralization BA.4、BA.5	SARS-CoV-2 Spike protein	iv	Not authorized by organization.	No	Sheridan et al. (75)

KTRs, kidney transplant recipients; SARS-CoV-2, Severe acute respiratory syndrome coronavirus 2; iv, IntraVenous; im, intramuscular injection; FDA:the United States Food and Drug Administration; EUA, Emergency-Use-Administration; COVID-19, Corona Virus Disease 2019.

## 4 Application of antiviral drugs in renal transplant patients diagnosed with Omicron

Antiviral medications play a vital role in helping renal transplant patients fend off SARS-CoV-2 infection. However, due to the ongoing mutations of SARS-CoV-2, the treatment approach for infected renal transplant patients is frequently adapted. Unlike previous variants, Omicron features mutations in both the RNA-dependent RNA polymerase (RdRp) and the primary protease of SARS-CoV-2. These are the specific targets of antiviral drugs such as RdRp inhibitors (such as remdesivir and molnupiravir) and the main protease inhibitor PF-07304814. Consequently, the effectiveness of current drugs against Omicron has been reduced (63). Therefore, it is crucial to identify the antiviral drugs beneficial for renal transplant patients. Current research suggests that antiviral compounds such as remdesivir and molnupiravir are effective against Omicron (32).

### 4.1 Remdesivir

Remdesivir was initially developed for treating hepatitis C and Ebola virus diseases. During the COVID-19 pandemic, remdesivir exhibited promising therapeutic effects against SARS-CoV-2 (64). Indeed, it is crucial to highlight that remdesivir can interact with cytochrome P450 3A4 substrates, such as tacrolimus and sirolimus, potentially leading to an increase in their blood concentrations. This interaction can have adverse effects on patients, especially in the presence of elevated inflammatory conditions (65), and potential kidney toxicity may also exist (66). Therefore, it is necessary to investigate whether remdesivir is suitable for use in KTRs.

A retrospective cohort study from China demonstrated that remdesivir treatment was safe and clinically feasible in KTRs (67). Similarly, Elec et al. (68) evaluated the effects of remdesivir on overall mortality, ICU mortality, and renal function outcomes in COVID-19 hospitalized patients who underwent kidney transplantation. The authors deduced that remdesivir did not exert any nephrotoxic effects on KTRs.

Remdesivir also exerts therapeutic effects on KTRs diagnosed with the Omicron variant. In Spain, remdesivir has been approved for treating severe pneumonia, respiratory failure, and COVID-19 patients who have had symptoms for less than 8 days. In non-hospitalized patients at high risk of COVID progression, the safety of a three-day course of remdesivir is acceptable, and the risk of hospitalization or death is 87% lower than that of placebo (69). In addition, a cohort study conducted at a clinical hospital in Barcelona, Spain, included all patients with comorbidities (including solid organ transplantation) in the remdesivir treatment cohort. The subsequent cohort study found that in the remdesivir-treated cohort of patients with the Omicron variant, severe illness could only be avoided by taking the drug early (within ≤7 days of symptom onset), and if the drug was administered correctly during the viremia phase of the disease, it was effective. Cacho et al. (70) also noted that remdesivir could be safely administered to KTRs with eGFR <30 mL/min. Therefore, remdesivir is an important and viable treatment option for KTRs diagnosed with the Omicron variant.

### 4.2 Molnupiravir

Molnupiravir is an orally administered antiviral medication that hinders the replication of SARS-CoV-2 by suppressing the RdRp, thereby exerting antiviral activity (71). Its *in vitro* effectiveness is approximately on par with that of remdesivir, and it has been proven to lower hospitalization rates compared with a placebo. A large-scale phase III clinical trial involving oral molnupiravir in COVID-19 patients showcased notable clinical advantages.

During the Omicron pandemic, KTRs treated with molnupiravir showed good clinical progress and a lower risk of hospitalization without any adverse effects. At the kidney level, molnupiravir exhibited good tolerance and no evidence of drug-induced nephrotoxicity or interaction with immunosuppressive therapy (72). Poznański et al. demonstrated through their experiments that early treatment with molnupiravir as a standalone therapy was safe and well-tolerated among a cohort of patients with ESKD who were either undergoing hemodialysis or had received a kidney transplant subsequent to SARS-CoV-2 infection (37, 73), and the Omicron variant was highly sensitive to molnupiravir (74, 75). Therefore, molnupiravir is one of the most beneficial drugs for KTRs diagnosed with the Omicron variant.

During the period when the Omicron subvariant BA.2.2 was prevalent in Hong Kong, early treatment with molnupiravir markedly lowered the risk of death in comparison to receiving no antiviral therapy (76). Therefore, we believe that clinical practitioners can consider molnupiravir as a treatment option for KTRs diagnosed with Omicron. The same study also indicated that the early administration of nirmatrelvir/ritonavir had comparable effects, and its use was correlated with a decreased risk of COVID-19-related hospitalization (76).

### 4.3 Nirmatrelvir/ritonavir

The combination of nirmatrelvir and ritonavir, marketed as Paxlovid by Pfizer, has demonstrated its effectiveness in treating COVID-19 patients and lowering the mortality rate among individuals with severe COVID-19 and those at high risk (77, 78). Nirmatrelvir is an effective inhibitor of the 3-chymotrypsin-like protease (3CLPro), also known as MPRO, in SARS-CoV-2, which plays a crucial role in the process of COVID-19 virus infection (79). Ritonavir, initially developed as an HIV protease inhibitor, slows down the metabolism of nirmatrelvir. It inhibits a specific enzyme, recombinant human CYP3A4 enzyme (CYP3A4), in the intestine, liver, and other body parts (80). In nirmatrelvir/ritonavir, ritonavir functions as an inhibitor of CYP3A4 to slow down the metabolism of nirmatrelvir (81).

The efficacy of nirmatrelvir/ritonavir against the Omicron variant of COVID-19 is a matter of concern. Recent *in vitro* studies have proven that nirmatrelvir retains its efficacy against all Omicron sub-lineages (82), and there have been numerous reports regarding the clinical effectiveness of nirmatrelvir/ritonavir in patients infected with the Omicron variant (83). Therefore, nirmatrelvir/ritonavir remains an important treatment option for patients diagnosed with the mysterious Omicron variant.

Although KTRs may benefit from nirmatrelvir/ritonavir (84, 85), the potential for this drug to interact with immunosuppressive drugs and harm patients cannot be overlooked (86). The interaction between nirmatrelvir/ritonavir and tacrolimus has been widely reported (87–89). A case report describes a 14-year-old renal transplant recipient maintained on tacrolimus and prednisone postoperatively, who received paxlovid upon infection with omicron, subsequently experiencing supra-therapeutic levels of tacrolimus and elevated serum creatinine (10.1007/s00467-022-05712-0). Concurrently, another report indicated that co-administration of paxlovid significantly reduced the clearance of tacrolimus to approximately 3.9% of the pharmacokinetic values in the normal population, with an inter-compartmental clearance (Q/F) decreased significantly to about 2.7% of the standard pharmacokinetic values. Thus, a significant drug–drug interaction exists between tacrolimus and paxlovid (10.1007/s40261-022-01180-4). Consequently, if paxlovid is administered, tacrolimus should be discontinued, and tacrolimus may be resumed when blood concentrations are within therapeutic range.

Because ritonavir is an inhibitor of CYP3A4, KTRs often use calcineurin inhibitors (CNIs), such as cyclosporine, tacrolimus, and mTOR inhibitors, including sirolimus and everolimus, which are highly dependent on CYP3A4 metabolism. Therefore, when CYP3A4 is exposed to ritonavir inhibition, the patient’s plasma drug levels increase significantly, increasing the risk of drug toxicity. Consequently, KTRs using nirmatrelvir/ritonavir require more frequent monitoring of blood drug concentrations (90). Although patients diagnosed with the Omicron variant may benefit from nirmatrelvir/ritonavir, these inhibitors are critical in preventing rejection reactions in KTRs, and drug interactions must be considered by clinical doctors when prescribing medication (72).

The clinical interaction between nirmatrelvir/ritonavir and immunosuppressive drugs can be effectively managed using standardized dosing schedules. In a single-center retrospective study of 14 KTRs treated with nirmatrelvir/ritonavir, the patients stopped taking tacrolimus 12 h before receiving nirmatrelvir/ritonavir treatment, were administered 20% of the cyclosporine dose, and continued taking the regular dose after completing nirmatrelvir/ritonavir treatment. In the subsequent follow-up, no deaths or viral pneumonia were observed in this study (84). A prior study conducted by Salerno et al. (91) demonstrated that when organ transplant patients adhered to a regimen involving the discontinuation of tacrolimus/mTOR inhibitors or a reduction in cyclosporine doses to 20% of the baseline daily dose over a five-day course of nirmatrelvir/ritonavir treatment, patients were able to prevent an elevation in drug concentration during the nirmatrelvir/ritonavir treatment period. Therefore, it is essential to develop standardized dosing schedules to effectively manage drug interactions and prevent harm caused by such interactions in patients.

If a patient still experiences toxicity symptoms during the use of nirmatrelvir/ritonavir, phenytoin (a CYP3A4 inducer) can be used to relieve these symptoms quickly. In the case of KTRs who develop acute tacrolimus toxicity after using nirmatrelvir/ritonavir, phenytoin can be used to rapidly decrease tacrolimus levels to within the therapeutic range. Moreover, no side effects of phenytoin (such as hypotension and arrhythmia) have been observed in KTRs (92), consistent with the case report results of Sindelar et al. (93).

### 4.4 Other antiviral drugs

During the COVID-19 pandemic, several antiviral drugs such as azvudine (94), ribavirin (95), and AT-527 (96) demonstrated effectiveness in treating COVID-19. However, their safety and efficacy in KTRs have not yet been characterized. In addition, SARS-CoV-1 3CLPRO and SARS-CoV-2 3CLPRO have approximately 96% amino acid sequence similarity and highly conserved active sites, suggesting that treatment agents developed for SARS-CoV-1 may also exhibit high *in vitro* efficacy against SARS-CoV-2 (97). Perhaps more antiviral drugs could be sought to help this special population avoid infection with Omicron.

In summary, in Omicron-affected areas, treatment guidelines for KTRs recommend remdesivir, molnupiravir, and nirmatrelvir/ritonavir. When compared with placebo, patients treated with nirmatrelvir/ritonavir exhibited the lowest risk of hospitalization or death (odds ratio [OR], 0.12; 95% confidence interval [CI]: 0.06–0.24), followed by remdesivir (OR, 0.13; 95% CI: 0.03–0.57), and then molnupiravir (OR, 0.67; 95% CI: 0.46–0.99) (98). Therefore, nirmatrelvir/ritonavir should be the first choice for doctors currently. However, due to the special status of KTRs, although nirmatrelvir/ritonavir can reduce the risk of severe COVID-19, its use in conjunction with other immunosuppressants increases drug concentration, and thus, the benefits and risks should be carefully weighed in clinical practice. Patients should use this medication under the guidance of a professional doctor, and healthcare professionals should exercise caution when assessing the prescription of nirmatrelvir/ritonavir for KTRs who are currently taking immunosuppressive drugs.

## 5 Vaccination for prevention of Omicron infection in KTRs

Since the emergence of the COVID-19 pandemic, researchers worldwide have worked on developing vaccines to boost population immunity and reduce SARS-CoV-2 infection risk. Large-scale vaccination efforts globally are seen as a highly effective strategy for achieving herd immunity against SARS-CoV-2 (99). During the Omicron outbreak, most people received third or fourth vaccine doses. However, it remains unclear whether COVID-19 vaccinations with the Omicron variant can effectively help KTRs, a question that remains both interesting and concerning.

### 5.1 Vaccination and KTRs

Vaccine administration was found to reduce the mortality rate among SARS-CoV-2-infected KTRs (100, 101). Mosconi et al. (102) also confirmed the efficacy of COVID-19 vaccines in managing the risk of infection and enhancing clinical outcomes in individuals with compromised immune systems.

Although KTRs can benefit from vaccination, previous research has shown that their humoral response to mRNA SARS-CoV-2 vaccines is reduced compared with individuals with normal immune function (103). Tauzin et al. (104) found that immunocompromised SOTRs had suboptimal efficacy following one or two vaccine doses during the COVID-19 pandemic. Moreover, another observational study found that administering the second dose of either the ChAdOx1 nCoV-19 (Oxford-AstraZeneca) or BNT162b2 (Pfizer/BioNTech) vaccine did not reduce the risk of infection (105). However, the third dose improved immunogenicity and reduced the risk of hospitalization after infection with Alpha, Beta, Gamma, and Delta variants (103). Therefore, the primary vaccination plan for transplant recipients is currently recommended to include three doses of the mRNA COVID-19 vaccine. Nevertheless, prior studies have not included the mid-term clinical outcomes of transplant recipients who have been vaccinated against the Omicron variant. This suggests a gap in our understanding of the mid-term effects of vaccination in this specific population (106).

### 5.2 Effects of the third vaccine on protection against Omicron in KTRs

The limited vaccine options for KTRs due to their unique immunosuppressed status make using a third dose of mRNA vaccine crucial. However, studies have shown that even after receiving three doses of mRNA vaccine, nearly half of the KTRs cannot produce sufficient levels of antibodies, thus failing to provide clinical protection (107). Therefore, it is imperative to thoroughly explore the effectiveness of administering a third vaccine dose in shielding KTRs from the potential threats posed by the Omicron variant.

The third dose of vaccine is beneficial in aiding KTRs to resist Omicron. Benning et al. (108) observed that KTRs significantly reduced levels of anti-S1 IgG, surrogate neutralization, and anti-receptor-binding domain antibodies compared with healthy controls after receiving the third dose of the vaccine. However, their ability to neutralize Omicron is lower than that of SARS-CoV-2 or other variants. Similarly, Panizo et al. (109) examined the impact of a third dose of a matching mRNA vaccine on SARS-CoV-2 RBD total antibody levels in a cohort of 21 KTRs. They detected RBD-specific antibodies in 19 of the 21 KTRs and identified neutralizing antibodies against the SARS-CoV-2, Delta, and Omicron variants, indicating the effectiveness of the third dose. Furthermore, while the neutralizing activity against Delta and Omicron variants in transplant patients remained low after receiving the third vaccine dose, the Fc-mediated effector function in SOTRs reached a level similar to that of a cohort of healthy individuals (104). Stich also indicated that while the standard mRNA vaccine regimen could weaken the humoral immune response in immunocompromised children and adolescents with kidney disease, reducing the vaccine dose could result in effective antibodies against Omicron in approximately 10% of patients (110). In addition, the study revealed that patients who did not develop humoral immune responses after the third vaccine dose either received an immunosuppressive regimen that included MMF or had an inherited T-cell defect disease.

Rodríguez-Cubillo et al. (111) also reported that compared with unvaccinated KTRs, vaccinated KTRs with at least low antibody titers (>20 AU/mL) exhibited improved outcomes in relation to pneumonia and mortality rates. Furthermore, patients with antibody titers >100 AU/mL had even better outcomes compared with those with lower antibody titers. Therefore, further research is required to explore how to improve the vaccine response in patients.

### 5.3 Optimizing vaccine response in KTRs

Although the third vaccine dose plays a certain role, the humoral and cellular responses of KTRs remain severely impaired after vaccination. Therefore, optimizing the vaccine response in KTRs should be the primary consideration.

#### 5.3.1 Using additional or alternative vaccination strategies is one way to optimize the immune response to vaccination in KTRs

Sanders et al. (112) observed the immune response of 267 KTRs who received the mRNA-1273 vaccine 6 months after transplantation and compared the same with a control group of 181 individuals. The study revealed that 6 months after vaccination, S1-specific antibodies were present in 100% of the control group, 98.7% of CKD G4/5 patients, 95.1% of dialysis patients, and 56.6% of KTRs. Most participants also possessed neutralizing antibodies against SARS-CoV-2 and the Delta variants, although neutralizing antibodies against the Omicron variant were notably scarce. In addition, S-specific T-cell responses were identified in 75.0% of the control group, 69.4% of CKD G4/5 patients, 52.6% of dialysis patients, and 12.9% of KTRs at the six-month mark, with T-cell responses notably lower at 6 months compared with the levels observed 28 days after vaccination. The interplay of low antibody levels, diminished T-cell responses, and the absence of neutralization against emerging variants implies that KTRs might necessitate supplementary or alternative vaccination approaches.

Hod et al. (113) reported that a fourth dose of BNT162b vaccine could effectively reduce the incidence and severity of Omicron in KTRs. Although Del Bello et al. (107) previously suggested that delaying the fourth dose of the vaccine might enhance vaccine immunogenicity in KTRs, they concluded that timely vaccination-induced antibody concentration increases still appear insufficient to provide clinical protection for KTRs, particularly against Omicron, where breakthrough infections occur even at very high concentrations. Presently, there is conflicting data from current studies regarding the efficacy of a fourth vaccine dose in KTRs. Nevertheless, administering a fourth vaccine dose represents another effort to enhance the immune response of KTRs to vaccination.

Chang et al. (114) found that the three-dose mRNA vaccine had limited effectiveness in eliciting antiviral antibodies against all viral strains in fully vaccinated, COVID-19-naïve SOTRs. Only 34.0% of patients exhibited detectable anti-RBD IgG and neutralizing activity against the Alpha, Beta, and Delta variants, and merely 8.5% of patients demonstrated additional neutralizing capacity against Omicron. In contrast, 79.5% of convalescent SOTRs who received two vaccine doses displayed high levels of anti-RBD IgG and neutralizing activity against Omicron. These findings underscore the substantial influence of prior infection on the evolution of the immune response to SARS-CoV-2 vaccination in SOTRs, emphasizing the necessity for alternative strategies to safeguard vaccine-hyporesponsive populations.

#### 5.3.2 Immunomodulatory strategies as one of the optimization methods for KTRs’ vaccination response

KTRs require immunosuppressants before and after transplantation to reduce the occurrence of immune rejection reactions. Studies have indicated that KTRs can significantly improve their existing vaccine protection efficacy by reducing or temporarily discontinuing the use of immunosuppressants (115). Therefore, the problem of a significant decrease in the serum conversion rate of KTRs after COVID-19 vaccination may be resolved by adjusting adaptive immunomodulatory strategies (116).

##### 5.3.2.1 Mycophenolic acid

Mycophenolic acid (MPA) is currently the most commonly used anti-proliferative drug in clinical practice. Kühn et al. (117) observed that the humoral response to vaccination was significantly stronger in KTRs who stopped MPA 1 month after vaccination. The transplant function remained stable in KTRs who discontinued MPA use during the study period, and no rejection reactions occurred in the patients. Therefore, cessation of MPA use may be related to enhanced allogeneic immune responses. However, this effect lasted only for 3 months because in this study, a general decrease in humoral immunity was observed in all KTRs 3 months after vaccination. Subsequently, Benninng et al. (116) demonstrated that short-term discontinuation of MPA could increase the antibody titers of KTRs after vaccination. These results showed that SARS-CoV-2-specific antibodies in individuals who discontinued MPA use during vaccination increased significantly. Simultaneously, in patients who discontinued MPA for a short time, the transplantation function and the level of free DNA from donor sources remained stable, indicating that the patient’s kidney transplant status was good, and there was no acute rejection reaction during the short-term follow-up period.

Frölke et al. (118) concluded that modifying MMF/MPA therapy prior to vaccination may enhance the vaccine response in KTRs who face a heightened risk of not developing antibodies after receiving the SARS-CoV-2 vaccine. Interestingly, Regele et al. (119) found that discontinuing MPA or azathioprine (AZA) for 2 weeks did not increase the serum conversion rate of KTRs who did not respond to the previous three to four doses of SARS-CoV-2 vaccine. However, the sample size of their study was small, and immunosuppression was discontinued only briefly for 2 weeks; accordingly, no conclusion can be drawn from this trial regarding the effects of MPA/AZA discontinuation on serum conversion rates.

##### 5.3.2.2 Mycophenolate mofetil

MMF, marketed under the brand name Cellcept, is a semi-synthetic derivative of MPA that was isolated from the fermentation broth of *Penicillium glaucum*. It is primarily used to prevent allograft rejection in organ transplantation (120).

Kantauskaite found that the likelihood of producing anti-SARS-CoV-2 antibodies in KTRs treated with immunosuppressive regimens that did not include MMF was 13-fold higher than that in KTRs treated with MMF-containing regimens. In KTRs treated with MMF, daily doses of MMF equal to or less than 1 g were associated with up to a five-fold increase in humoral immune response to the vaccine compared to KTRs treated with higher doses of MMF (121). Hence, MMF utilization is associated with a weakened immune response to SARS-CoV-2 vaccination in KTRs, and adjusting the MMF dosage is a viable option to enhance the humoral immune response.

In a multicenter observational study, the impact of the third SARS-CoV-2 vaccine on KTRs who were previously seronegative was analyzed. The study focused on temporarily reducing the MMF dose among propensity-matched KTRs. Among 174 previously seronegative KTRs, 56 (32%) became seropositive after the third dose, while only three KTRs developed neutralizing antibodies against the Omicron variant. The researchers concluded that reducing the MMF dose by 33% or more before vaccination is a promising strategy to enhance the effectiveness of SARS-CoV-2 vaccination in KTRs (121). Another study demonstrated that immunosuppressive regimens, excluding MMF, along with lower doses of calcineurin inhibitors and the incorporation of everolimus, were correlated with an increased rate of humoral response in elderly KTRs following COVID-19 vaccination (122). Stumpf et al. (123) also indicated that MMF/MPA is the chief mediator responsible for the delayed humoral response and decreased antibody decline in KTRs after SARS-CoV-2 mRNA vaccination. Although suspension of MMF may increase the immune response to SARS-CoV-2 vaccination, further exploration is required to ensure serum conversion.

##### 5.3.2.3 Belatacept

CNIs can bind to calcineurin and inhibit its activity, thereby exerting immunosuppressive effects. Cyclosporine and tacrolimus are drugs commonly used in KTRs (124). Compared with CNIs, belatacept can significantly reduce the production of DSA during the transplantation process, improve the survival rate of transplants, and maximize the avoidance of CNI nephrotoxicity (125). Therefore, some immunosuppressive regimens gradually favor the use of belatacept.

Amid the COVID-19 pandemic, treating SOTRs with belatacept diminished the immune responses to SARS-CoV-2 mRNA vaccination. It is recommended that the vaccine be administered separately from belatacept infusion to increase immunogenicity (126). In a study by Wiedemann et al., 52 KTRs were observed, with 31 receiving CNIs treatment and 21 receiving belatacept treatment. After two vaccine doses, 46.9% of patients generated anti-S IgG. Among them, 21.4% in the belatacept group produced anti-spike IgG, while 83.3% in the CNIs group did. Moreover, T-cell function was notably weaker in the belatacept group compared with the CNIs group. This indicates that belatacept-based regimens significantly reduce both humoral and cellular vaccine responses in transplant patients. From a clinical standpoint, it is crucial to assess IgG responses in transplant recipients on belatacept-based regimens and devise appropriate preventive or post-exposure strategies (127, 128). Chavarot et al. (129) found that with the emergence of the third dose of the BNT162b2 mRNA COVID-19 vaccine, the antibody response of KTRs receiving belatacept treatment with the three doses of mRNA vaccine was weaker. The study by Morena et al. (130) revealed that factors related to the decrease in anti-SARS-CoV-2 antibody response after mRNA vaccination in belatacept KTRs may include the use of prednisone, age ≥ 60 years, glomerular filtration rate < 45 mL/min/1.73 m^2^, and no history of SARS-CoV-2 infection.

In summary, the distinctive traits of KTRs result in their limited response to vaccination. Therefore, enhancing vaccine potency is crucial to maximize their vaccine benefits. Modifying vaccine administration strategies during clinical care can boost KTRs’ immune response to SARS-CoV-2 vaccination. Additionally, further research is needed to ascertain the impact of reducing immunosuppressive drugs on post-vaccination immunity while managing the risk of rejection during vaccination-related changes in immunosuppression.

## 6 Conclusion and perspectives

The COVID-19 pandemic has lasted nearly 4 years, causing significant impacts on global health and the economy. The virus still threatens human health and life, and the current situation remains uncertain as infection rates fluctuate. KTRs are especially vulnerable to COVID-19 due to their extensive immunosuppressive treatment, which weakens their immune system and leads to added complications. Their reduced immunity leaves them at increased risk for COVID-19, particularly in the face of the ongoing transmission of the Omicron variant. In the next phase of the pandemic, it is crucial to prioritize developing and implementing effective preventive and control measures for those with potential immune deficiencies.

People with weakened immune systems have a reduced ability to produce protective antibodies, and mAbs and antiviral drugs can offer added protection against COVID-19. While sotrovimab, bebtelovimab, and tixagevimab/cilgavimab are suitable for KTRs, their effectiveness has waned with further Omicron mutations. Therefore, more research is warranted to develop new mAbs or mAb combinations for COVID-19 prevention and treatment in KTRs, offering safe, long-lasting protection in addition to vaccination. Although antiviral drugs can also benefit KTRs, they may interact with other immunosuppressive drugs, potentially increasing drug levels in the blood. Hence, clinical guidelines should be updated to address this trade-off.

As immunosuppressed individuals, KTRs are at higher risk of severe COVID-19. Thus, timely vaccination is crucial for them despite potentially reduced vaccine response. Vaccination has been proven to significantly decrease COVID-19 severity and mortality. Therefore, it is advisable to urge KTRs in stable post-transplantation phases to get vaccinated against COVID-19.

Additionally, with the ongoing emergence of new virus variantssuch as XBB and its lineage EG.5, which can evade host immune defenses, developing next-generation vaccines and alternative therapeutic approaches, including passive immunization, post-exposure prophylaxis, and cellular therapy, has gained heightened significance to enhance safeguarding against vaccine inadequacy. Additionally, considering the measurement of spike antibody titers and serum neutralization as routine indicators of vaccine response, mAbs, alternative vaccine regimens, or a reduction in immunosuppression could be selectively provided for KTRs. Adjusting immunosuppressive therapy strategies may improve vaccine responsiveness. However, it also exposes the potential risks of acute or chronic rejection. Therefore, reducing or temporarily stopping immunosuppressive treatment policies remains a dilemma when it comes to improving humoral responses.

Currently, the global COVID-19 situation remains uneven, with some regions experiencing low numbers of new cases, whereas others continue to persist. Many countries experienced repeated peaks during the epidemic. In the future, vulnerable populations, such as the elderly and those with weakened immunity or underlying conditions, must be the prime focus of prevention and control. Vaccines, specific drugs, and social distancing are effective measures against Omicron. As a susceptible population, KTRs require greater attention and effort to protect themselves. Further research is crucial to establish appropriate prevention and treatment guidelines.

## Author contributions

ZZ: Conceptualization, Data curation, Investigation, Supervision, Validation, Visualization, Writing – original draft, Writing – review & editing. HS: Conceptualization, Funding acquisition, Investigation, Supervision, Writing – original draft. XH: Conceptualization, Validation, Visualization, Writing – review & editing. ZX: Conceptualization, Validation, Writing – review & editing. MF: Writing – review & editing. YB: Visualization, Writing – review & editing. YD: Visualization, Writing – review & editing. BL: Visualization, Writing – review & editing. XS: Validation, Writing – review & editing. JZ: Conceptualization, Project administration, Writing – review & editing. CS: Investigation, Project administration, Supervision, Writing – original draft, Writing – review & editing.

## Glossary

AKI: acute kidney injury
AZA: azathioprine
ACE2: angiotensin converting enzyme 2
COVID-19: corona virus disease 2019
CKD: chronic kidney disease
CRS: cytokine storms
CNIs: calcineurin inhibitors
CYP3A4: recombinant Human CYP3A4 Enzyme
ESRD: end-stage renal disease
EUA: emergency use administration
FDA: the United States food and drug administration
IL-1β: interleukin-1β
IL-6: interleukin-6
KTRs: kidney transplant recipients
MPA: mycophenolic acid
MMF: mycophenolate mofetil
MHRA: the UK Medicines and Healthcare products regulatory agency
RRT: renal replacement therapy
RBD: receptor-binding domain
RdRp: RNA-dependent RNA polymerase
SARS-CoV-2: severe acute respiratory syndrome coronavirus 2
SOTR: the solid organ transplant recipients
TNF: tumor necrosis factor
WHO: the world health organization
3CLPro: chymotrypsin-like protease
